# Functional biomaterials for the diagnosis and treatment of peritoneal surface malignancies

**DOI:** 10.1002/SMMD.20230013

**Published:** 2023-07-13

**Authors:** Xiusen Qin, Mingli Su, Huili Guo, Binying Peng, Rui Luo, Junwen Ye, Hui Wang

**Affiliations:** ^1^ Department of General Surgery The Sixth Affiliated Hospital Sun Yat‐sen University Guangzhou China; ^2^ Guangdong Institute of Gastroenterology Guangdong Provincial Key Laboratory of Colorectal and Pelvic Floor Diseases Biomedical Innovation Center The Sixth Affiliated Hospital Sun Yat‐sen University Guangzhou China; ^3^ Institute of Biomedical Innovation and Laboratory of Regenerative Medicine and Biomaterials Biomedical Material Conversion and Evaluation Engineering Technology Research Center of Guangdong Province Guangzhou China; ^4^ Department of Endoscopic Surgery The Sixth Affiliated Hospital Sun Yat‐sen University Guangzhou China; ^5^ Department of Infectious Diseases The Third Affiliated Hospital Sun Yat‐sen University Guangzhou China; ^6^ Zhongshan School of Medicine Sun Yat‐sen University Guangzhou China

**Keywords:** biomaterial, diagnosis, peritoneal surface malignancy, treatment

## Abstract

Peritoneal surface malignancies (PSM) can originate from tumors in many organs and are highly malignant, and difficult to diagnose and cure, posing a serious threat to the survival of patients. Although the diagnosis and treatment of PSM have made significant progress in the past two decades, numerous challenges remain. Recently, functionalized biomaterials have shown promise for PSM diagnosis and treatment. Hence, we review the progress of functionalized biomaterials for the diagnosis and treatment of PSM. We first introduce the classification and pathogenesis of PSM. We then discuss the applications of functionalized biomaterials for the diagnosis and treatment of PSM. In particular, we focus on functionalized biomaterials as drug carriers for the treatment of PSM, including chemotherapy, immunotherapy, targeted therapy, combination therapy, and other therapies. Finally, we summarized the current challenges and provided a perspective on the diagnosis and treatment of PSM.

## Graphical abstract

In recent years, functionalized biomaterials for peritoneal surface malignancies (PSM) have been widely studied. In this article, Wang and coworkers reviewed the progress of functionalized biomaterials for the diagnosis and treatment of PSM.

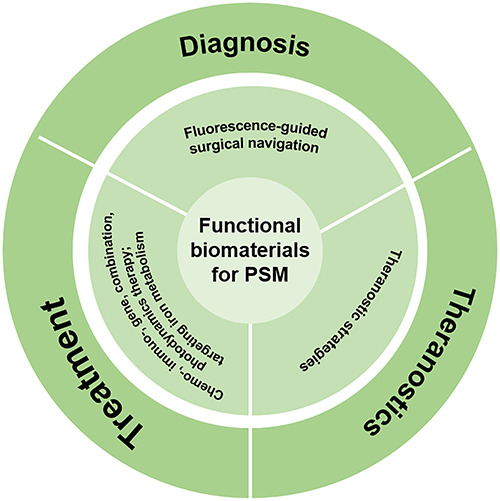

**Key points**

- Peritoneal surface malignancies (PSM) can originate from tumors in many organs, which have poor prognoses and are difficult to diagnose.
- Functional biomaterials for the diagnosis and treatment of PSM are discussed.
- Future research directions and current challenges of functional biomaterials for PSM have been prospected.

## 1 INTRODUCTION

**FIGURE 1:**
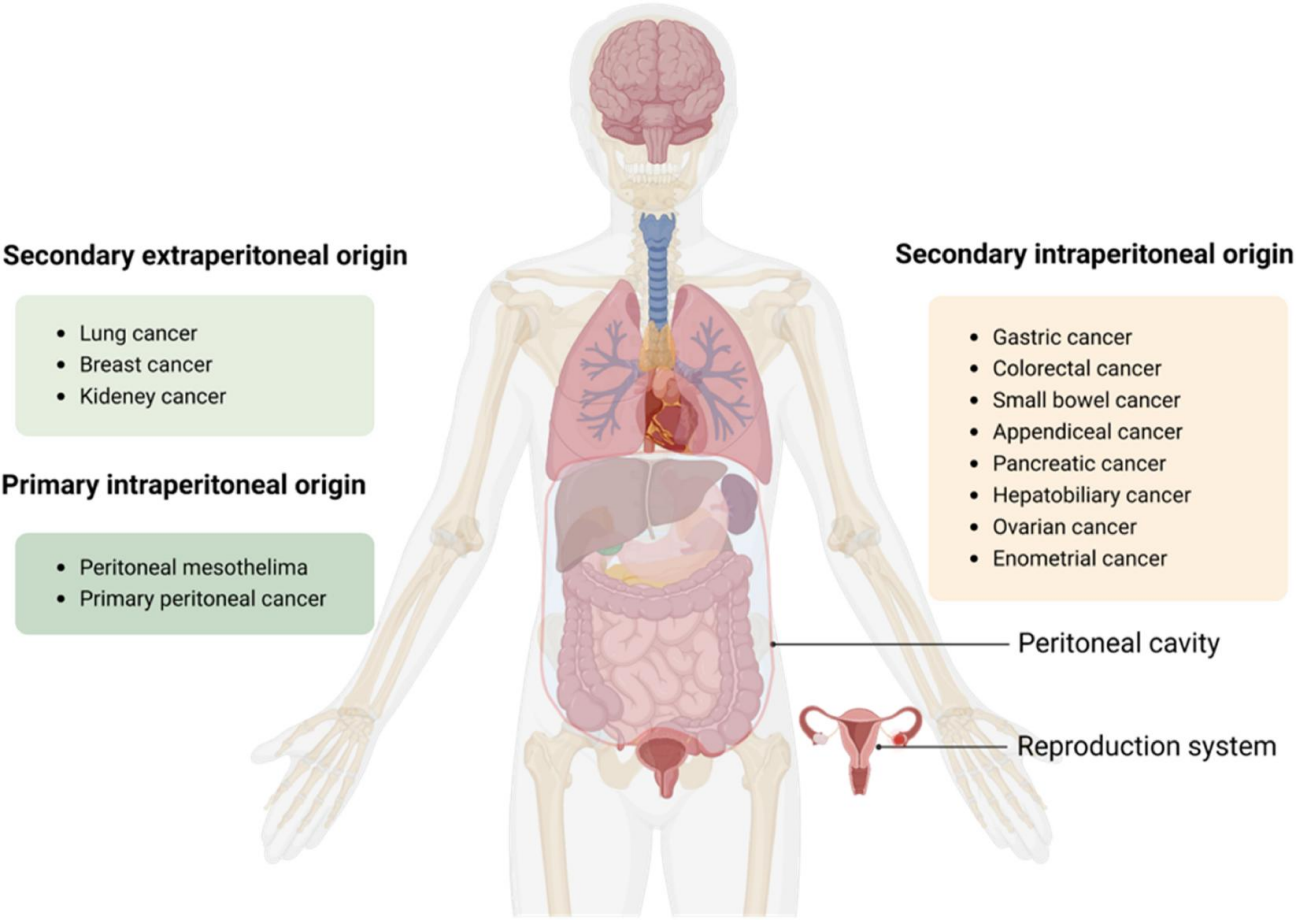
Classification of peritoneal surface malignancies (created with BioRender.com).

Peritoneal surface malignancies (PSM) are malignant tumors occurring on the peritoneal surface, including primary peritoneal tumors (e.g. primary peritoneal carcinoma and peritoneal mesothelioma) and secondary peritoneal tumors (e.g. peritoneal metastases from intraperitoneal and extraperitoneal tumors) (Figure 1).^1^ The specific incidence of PSM depends on the origin of the malignant tumor. Generally, primary PSM has a relatively low incidence, whereas secondary PSM, often from colorectal or gastric cancer, has a high incidence. For colorectal cancer, synchronous peritoneal metastasis occurs in 4%–15% of patients,^2^, ^3^ and the metachronous peritoneal metastasis are about 25%.^2^, ^4^ Gastric cancer has a higher incidence of peritoneal metastasis in gastrointestinal malignancies, ranging from 15% to 43% (including synchronous and metachronous peritoneal metastasis).^5^, ^6^ Moreover, the relative incidence of ovarian cancer peritoneal metastasis has been reported to be as high as 60%–70%.^7^ Compared with intraperitoneal tumors, extraperitoneal tumors are less likely to cause PSM.^8^

PSM has always been considered complicated diseases. On the one hand, small peritoneal tumors are widely distributed in the abdominal cavity, which are often difficult to remove completely because of the poor contrast between healthy and tumor tissue.

Residual cancer cells or lesions are the leading cause of postoperative recurrence.^9^ Of note, fluorescence‐guided surgical navigation has been considered a promising method for removing these small tumor nodules in recent years.^10^, ^11^ On the other hand, systemic chemotherapy has limited effects on PSM due to their inherent poor blood supply. In the last two decades, significant innovations in the treatment of PSM include cytoreductive surgery and intraperitoneal chemotherapy. The former is dedicated to removing all visible peritoneal tumors, and the latter is mainly for eliminating microscopic residual lesions. The application of cytoreductive surgery plus intraperitoneal chemotherapy significantly prolonged the overall survival of PSM patients.^12^, ^13^, ^14^ For example, the median overall survival of PSM patients with colorectal cancer is less than 12.6 months in the early stage.^15^ While it has been up to 41.7 months in recent years.^16^ However, there are still many problems with the treatment of PSM. Surgical treatment dilemmas include the existing high recurrence rate after surgical resection and unresectable tumor nodules at specific sites.^17^ Intraperitoneal chemotherapy can remove residual tumor cells or lesions to a certain extent, but it can also cause complications such as intestinal obstruction and anastomotic leakage.

In recent years, functionalized biomaterials have been widely used for the diagnosis and treatment of PSM. Researchers are focusing on the development of near‐infrared fluorescent materials with targeted functions for fluorescence‐guided surgical navigation.^18^, ^19^, ^20^, ^21^ and the construction of drug delivery systems for intraperitoneal treatment.^22^, ^23^, ^24^ In this paper, we reviewed the pathogenesis mechanism of PSM and then summarized the latest progress of materials science in the diagnosis and treatment of PSM. In the end, we offer some perspectives on current challenges and future development.

## 2 PATHOGENESIS OF PERITONEAL SURFACE MALIGNANCIES

The peritoneum consists of a layer of mesothelial cells supported by a basement membrane and a layer of connective tissue, which is a network of lymphatic and blood vessels. The fibrous connective tissue layer also contains collagen, adipose tissue, lymphocytes, fibroblasts, and macrophages. The peritoneal cavity is a potential space consisting of the parietal and visceral peritoneum. The former covers the abdominal wall and pelvic cavity, and the latter covers most of the intra‐abdominal organs. About 50 mL of fluid is in the peritoneal cavity, consisting of water, immune cells, electrolytes, antibodies, and other components. The physiological functions of the peritoneum include lubrication, defense, repair, and absorption.^25^, ^26^

As with other solid tumors, PSM consists of cellular and non‐cellular components in which malignant cell populations are embedded. The cellular components include blood vessel cells, immune cells, and mesenchymal cells. Non‐cellular elements consist of extracellular vesicles, lipids, extracellular matrix, proteins and soluble metabolites, cytokines, chemokines, and growth factors.^27^

**FIGURE 2:**
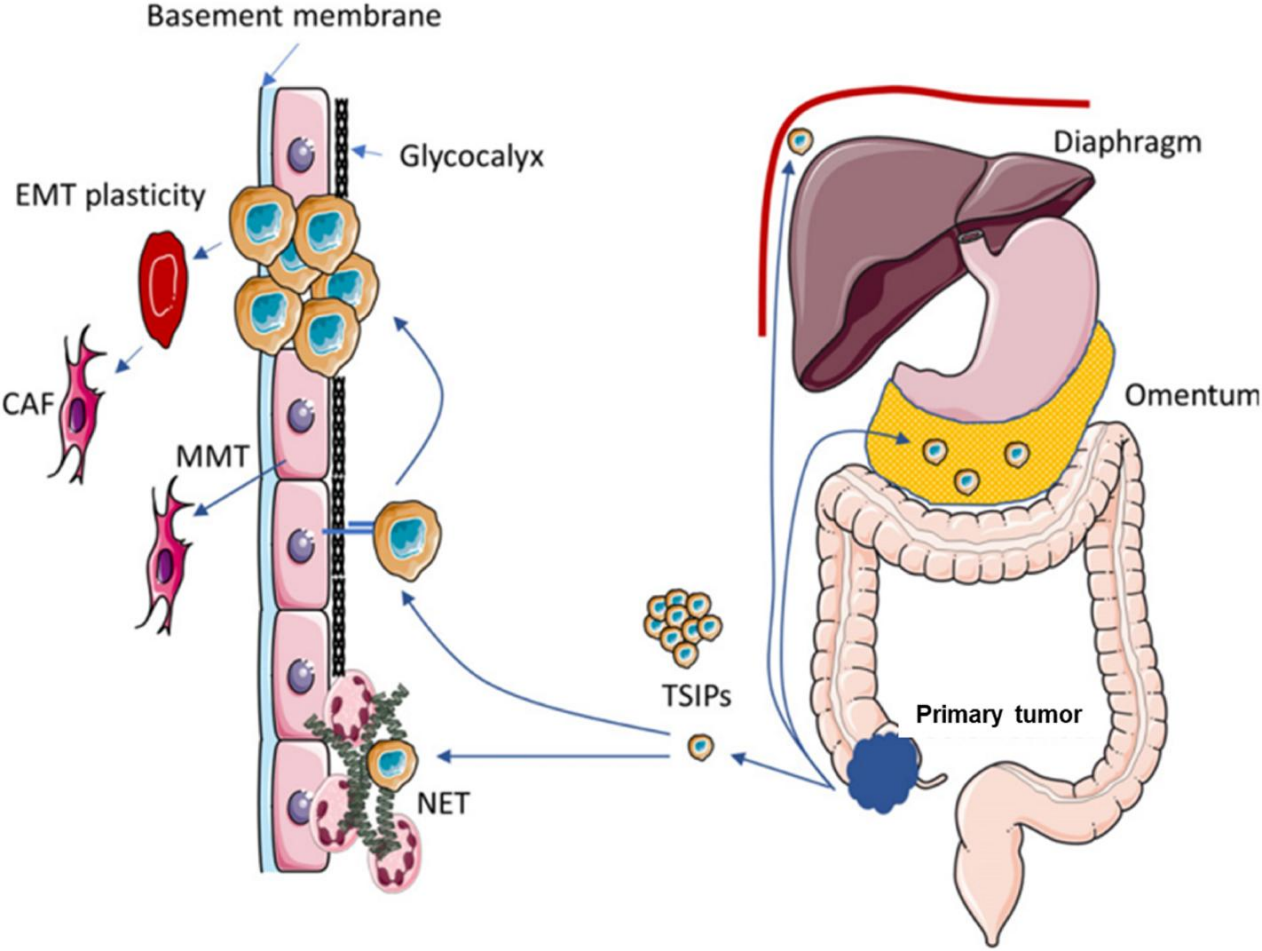
Pathogenesis of peritoneal surface malignancies.^27^ (Reprinted with permission. Copyright 2020, Elsevier.) Tumor cells shed from the primary tumor or as TSIPs and then adhere to mesothelial cells. Once the mesothelial layer is breached, EMT plasticity and MMT start. EMT, epithelial‐to‐mesenchymal; MMT, mesothelial‐to‐mesenchymal transition; TSIP, tumor spheres with inverted polarity.

The development of PSM can be generalized as a stepwise process beginning with the entry of malignant cells into the peritoneal cavity (Figure 2). (1) Cell shedding from the primary tumor. This can occur spontaneously in locally progressive and perforating tumors or due to iatrogenic causes.^28^, ^29^ The pathophysiology of peritoneal metastases from extraperitoneal tumors is not entirely clear, but it may relate to lymphatic and/or vascular pathways. (2) Intraperitoneal transport of tumor cells. Free tumor cells move passively in the abdominal cavity by gravity and diaphragm offset. Hence, cells usually follow a typical path, starting from the pelvis and following the right para‐colonic sulcus into the subdiaphragmatic space.^30^ (3) Mesothelial adhesion. Free tumor cells adhere to the mesenchymal or extracellular matrix layer by specific adhesion molecules, such as PECAM1, ICAM1, and VCAM.^31^ Mesenchymal damage can exacerbate the interaction, in which the integrity of cell junctions is lost, exposing the underlying basement membrane. Alternatively, infection or surgery can increase the expression of adhesion molecules due to the inflammatory stimuli.^32^ (4) Invasion of the submesothelial layer. In areas of mesothelial cell contraction or peritoneal discontinuity, loose cancer cells enter the mesenchymal tissue. In addition, tumor cells can induce apoptosis of mesothelial cells.^33^ Once the mesothelial barrier is disrupted, tumor cells degrade the matrix by secreting matrix metalloproteinases. Besides, tumor cells can induce epithelial interstitial transformation, which leads to peritoneal fibrosis.^34^

## 3 FUNCTIONAL BIOMATERIALS IN PSM DIAGNOSIS

The symptoms of PSM are not specific, and their diagnosis mainly depends on imaging examinations and surgical explorations. Early diagnosis of PSM is of great significance in improving the survival of patients, but current imaging examinations are limited in detecting small peritoneal metastasis nodules.^35^ On the other hand, recent studies have shown that complete resection of peritoneal metastasis significantly improved overall survival.^13^, ^36^, ^37^ However, healthy and tumor tissue do not contrast well visually, so achieving effective cytoreductive surgery remains challenging.^38^ Therefore, it is of great value to develop functional biomaterials for the early diagnosis and intraoperative visualization of peritoneal metastasis.

### 3.1 Fluorescence‐guided surgical navigation

**FIGURE 3:**
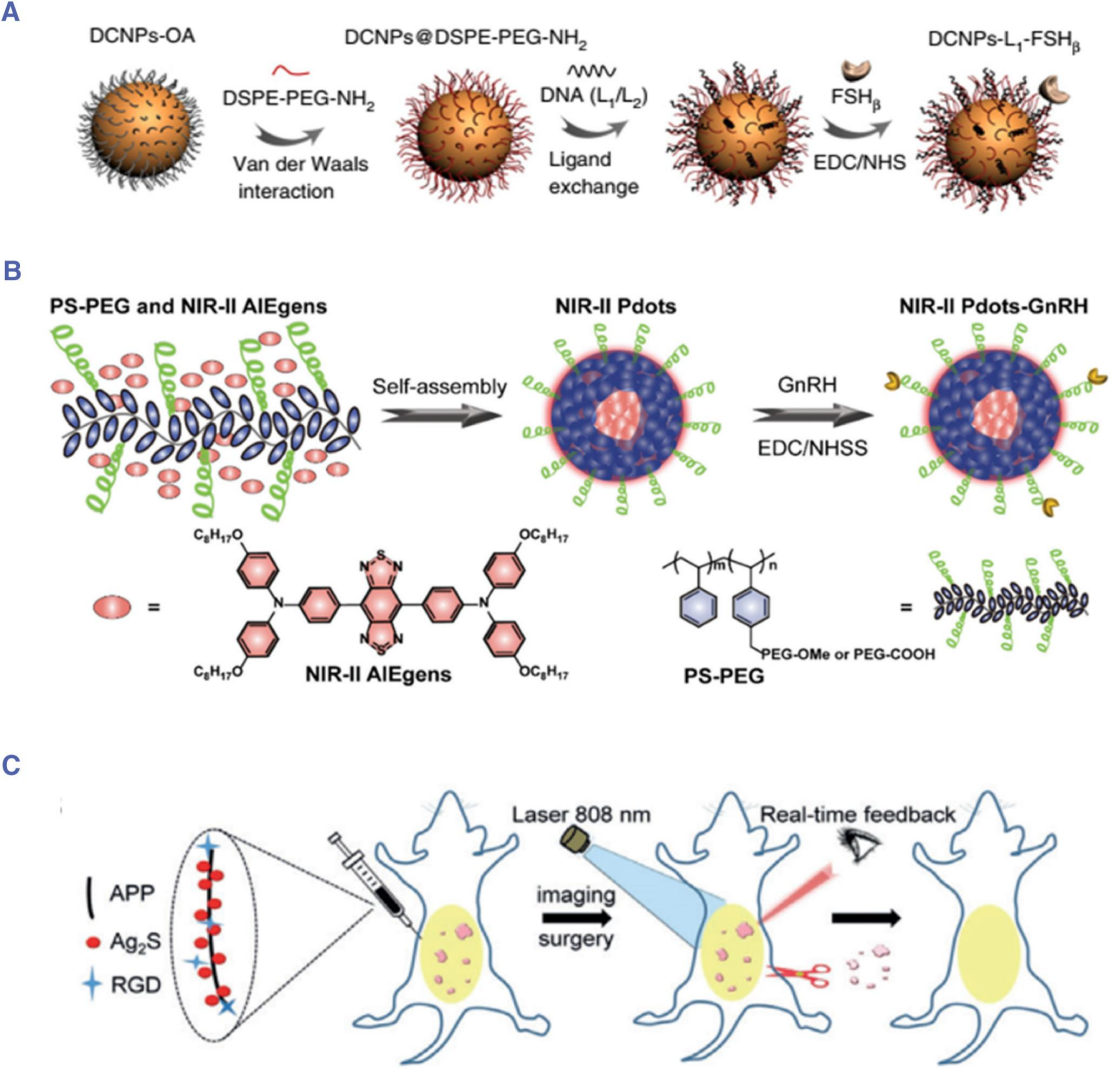
NIR‐II fluorescent‐guided surgical navigation for detection of peritoneal metastasis. (A) Schematic illustration of DNA and FSH_β_ modified DCNPs for detecting peritoneal metastasis of ovarian cancer.^19^ (Reprinted under the terms of the Creative Commons CC BY license. Copyright 2018, The Authors, published by Springer Nature.) (B) Schematic illustration of the preparation of NIR‐II polymer dots for targeting metastatic ovarian cancer.^21^ (Reprinted under the terms of the Creative Commons CC BY license. Copyright 2021, The Authors, published by John Wiley and Sons.) (C) Schematic illustration of the NIR‐II fluorescent nanochain probe for peritoneal carcinomatosis from glioblastoma.^18^ (Reprinted with permission. Copyright 2019, John Wiley and Sons). DCNPs, downconversion nanoparticles.

Fluorescence‐guided surgical navigation is a promising method for removing tumor nodules due to its characteristics of real‐time imaging, high contrast, and no ionizing radiation (Figure 3). In the last decade, many researchers have made great efforts to develop different fluorescent probes for fluorescence‐guided surgical navigation. Some probes have been designed for peritoneal metastasis imaging.^38^, ^39^ The emission wavelength of early fluorescent probes is in the visible (400–700 nm) or first near‐infrared (NIR‐I, 700–950 nm) region.^40^, ^41^, ^42^ These probes have limited penetration depth and spatial resolution (∼mm), as well as high tissue autofluorescence, which significantly reduces the efficiency of surgical treatments of tumors. In recent years, NIR‐II fluorescent probes (1000–1700 nm), such as carbon nanotubes,^43^ quantum dots,^44^, ^45^ rare earth doped nanoparticles,^19^, ^46^, ^47^, ^48^ and organic dyes,^49^, ^50^, ^51^, ^52^ have been ideal candidates for surgical navigation for their deep penetration depth (∼cm), high spatial resolution (∼μm), and very low tissue autofluorescence. Another critical aspect to consider for fluorescence‐guided surgery is a specific tumor targeting. Utilizing the tumor microenvironment characteristics and tumor‐specific receptors to develop targeted probes is an active area of research.

Ovarian cancer has some specific targets, such as follicle‐stimulating hormone (FSH) receptor and gonadotropin‐releasing hormone (GnRH) receptor. Therefore, many researchers focus on developing fluorescent nanoprobes for tracing peritoneal metastasis of ovarian cancer. Daisuke Asanuma et al. developed a targeting fluorescent probe based on the feature of high expression of *β*‐galactosidase in ovarian cancer cells for peritoneal metastasis of ovarian cancer.^38^ Specifically, they used a derivative of hydroxymethylrhodol (HMR) and *β*‐galactose to synthesize a fluorescent probe (HMRef‐bGal) that could penetrate the cell membrane of ovarian cancer cells and then it dissociates into HMRef in the presence of *β*‐galactosidase for fluorescence imaging. They tested the function of the probe on peritoneal metastasis models constructed from seven different kinds of ovarian cancer cell lines, showing that the probe could detect peritoneal metastatic nodules less than 1 mm in diameter. Wang et al. modified downconversion nanoparticles (DCNPs) with complementary DNA strands and follicle stimulating hormone peptide (FSH_β_).^19^ DCNPs‐L_1_‐FSH_β_ and DCNPs‐L_2_‐FSH_β_ were constructed, respectively. First, DCNPs‐L_1_‐FSH_β_ nanoprobes were injected through the tail vein of mice. After 8 h of liver and kidney metabolism, the nanoprobes were enriched only at the sites of ovarian metastasis. Then, DCNPs‐L_2_‐FSH_β_ nanoprobes were injected, and they could autonomously load with DCNPs‐L1‐FSH_β_ as DNA‐conjugated nanoprobes (Figure 3A). The results showed that the self‐assembled nanoprobes in mice can achieve a longer retention time at the sites of metastatic ovarian tumor and achieve better NIR‐II bioimaging.^19^ Similarly, Zhou et al. prepared NIR‐II polymer (NIR‐II Pdots) dots by self‐assembling NIR‐II aggregation‐induced emission luminogens and poly (styrene)‐graft‐poly (ethylene glycol) (PS‐PEG) in water.^21^ They further modified the NIR‐II Pdots with GnRH peptide (Figure 3B). The constructed nanoprobe not only has a high quantum yield but can also be used to remove the peritoneal and lymph node metastases of ovarian cancer with a diameter of about 2 mm under the navigation of NIR‐II fluorescent probes.

However, NIR‐II nanoprobes targeting peritoneal metastasis derived from colorectal cancer or other tumor types have rarely been reported. The possible reason is that most tumors lack specifically targeted receptor. Previous studies showed that arginine‐glycine‐aspartate (RGD) peptide can bind to integrin receptors on the surface of tumor cells.^53^, ^54^ Wang et al. developed a nanochain probe (APP‐Ag_2_S‐RGD) capable of producing NIR‐II fluorescence for intraoperative sensitive detection of peritoneal metastasis originating from U87‐MG glioblastoma cells by self‐assembling amphiphilic peptides (APP) into nanochains and then chemically cross‐linking Ag_2_S quantum dots and RGD peptides to them (Figure 3C).^18^ It can aid to clear avascular tumor metastasis with a minimum diameter of 0.2 mm under the guidance of the probe. Similarly, Guo and his colleagues showed a FeGd‐based nanoparticle for the imaging of gastric cancer with peritoneal metastasis, which loaded indocyanine green (ICG)/glucose oxidase (GOx) with conjugation of an RGD dimer (RGD2) and acid‐labile polymer mPEG.^55^ RGD2 is used to target tumors. Their results show that the probe can help detect tumor nodules with a minimum diameter of 3 mm. Its performance is not better than that of the previous study,^18^ possibly because ICG has a lower spatial resolution. The role of RGD‐based probes in other tumor‐derived peritoneal metastases should be verified by further studies.

In general, there is still much space for research in fluorescence‐guided surgical navigation. Future research should not only focus on the development of new fluorescent materials but also focus on the research progress of tumor microenvironments and specific targets of tumor cells to develop fluorescent probes with better targeting ability.

### 3.2 Others

Immuno‐positron emission tomography (PET) combines the tumor‐targeting function of monoclonal antibodies with the inherent sensitivity of PET technology, which is a potentially paradigm‐changing approach to molecular imaging.^56^ For example, Jeger et al. have shown that immunoconjugates showed higher tumor uptake in a human ovarian carcinoma peritoneal metastasis model.^57^ Immuno‐PET, as a non‐invasive imaging technique, can reveal the biological distribution and expression of tumor antigens, which can help in the diagnosis, treatment selection, patient stratification and treatment response prediction of PSM, thus assisting in clinical decision‐making.

## 4 FUNCTIONAL BIOMATERIALS IN PSM TREATMENT

Treatment modalities for PSM include cytoreductive surgery, systemic chemotherapy, and hyperthermic intraperitoneal chemotherapy (HIPEC). In addition, targeted drugs and immunotherapies are widely used to treat widespread metastatic diseases.^58^ Unlike liver and lung metastasis, peritoneal metastasis is characterized by a lack of blood supply, so the effect of systemic chemotherapy is limited.^9^ With the development of technology, HIPEC has become the primary selection of intraperitoneal chemotherapy. HIPEC, as a way of local‐regional therapy, plays a vital role in PSM treatment, which can increase the local drug concentration of the peritoneal cavity. Previous studies have confirmed that hyperthermic therapy and chemotherapy have synergistic effects.^13^ However, HIPEC still faces many challenges. Firstly, small molecule drugs and hydrophilic drugs applied to the abdominal cavity can be quickly absorbed into the blood circulation, resulting in blood or systemic side effects.^59^ Second, high molecular weight or hydrophobic drugs cause local toxicity and dose‐limiting effects.^60^ Besides, HIPEC also has many side effects, including bleeding, hemotoxicity, and anastomotic leak.^61^, ^62^ Therefore, for a long time, researchers have focused on developing drug delivery systems to improve the efficacy of intraperitoneal chemotherapy and reduce side effects. The materials used in the sustained release of intraperitoneal chemotherapy drugs mainly include particles (microparticles, nanoparticles, micelles, macromolecules, liposomes, etc.) and degradable hydrogel‐based sustained‐release materials.^63^

### 4.1 Chemotherapy

In the past decade, many researchers have focused on using drug‐loaded hydrogels to treat peritoneal metastasis. Injectable and thermosensitive hydrogels are ideal materials for intraperitoneal chemotherapy drug delivery due to their in situ gelling properties. The feature of temperature‐sensitive hydrogels is the co‐existence of hydrophilic and hydrophobic groups, which have the lowest critical dissolution temperature (LCST). When the temperature is lower than LCST, the hydrogel exists in a liquid state, and conversely, the material changes from a liquid form to a solid state. The most widely studied thermo‐sensitive hydrogels include poly(N‐isopropyl acrylamide) (PNIPAAm) and block copolymer‐based materials.^64^, ^65^, ^66^, ^67^, ^68^ Wang et al. used triblock copolymer PEG‐PCL‐PEG to load 5‐FU and realized the sustained release of 5‐FU in the intraperitoneal cavity.^69^ The results showed that the 5‐FU loaded hydrogel system could better inhibit the peritoneal dissemination of colorectal cancer cells in the intraperitoneal cavity of mice than the 5‐FU solution. Chung et al. synthesized a temperature‐responsive hydrogel through biodegradable ester linkage between the hydroxyl group of pluronic (PLU) and the carboxyl group of linoleic acid (CLA) as a drug delivery system for the controlled release of docetaxel for the peritoneal metastasis of gastric cancer.^70^ Intraperitoneal injection of docetaxel‐PLU‐CLA hydrogel can prolong survival in a mice model of peritoneal metastasis with gastric cancer cells, which indicated that PLU‐CLA can be a potential carrier for hydrophobic docetaxel in treating gastric cancer.

Of note, hydrogels can realize sustained release to a certain extent, but they exist in burst release due to the small molecular weight of chemotherapy drugs. Hence, some researchers have wrapped drugs in microspheres or nanoparticles and then mixed the microspheres into a hydrogel.^71^

### 4.2 Immunotherapy

In the era of immunotherapy, the extent of infiltration and composition of stromal immune cells are of increasing concern. For example, extensive immune cell infiltration (about 15%) is observed in colorectal cancer patients with microsatellite instability, leading to an excellent response to immune checkpoint inhibitor therapy.^72^, ^73^ Immunotherapy can kill cancer cells by modulating and stimulating the body's immune function. Strategies for cancer immunotherapy include immune checkpoint inhibitors, tumor vaccines, cytokines, adoptive cell therapy, antibody‐drug coupling, costimulatory receptor agonists, etc.^74^ The peritoneal cavity contains a large number of immune cells. It is an auspicious treatment method to activate the immune response of the peritoneal cavity to treat PSM.^27^ In this section, we summarize the application of immunotherapeutic agents‐loaded hydrogels in PSM treatment.

**FIGURE 4:**
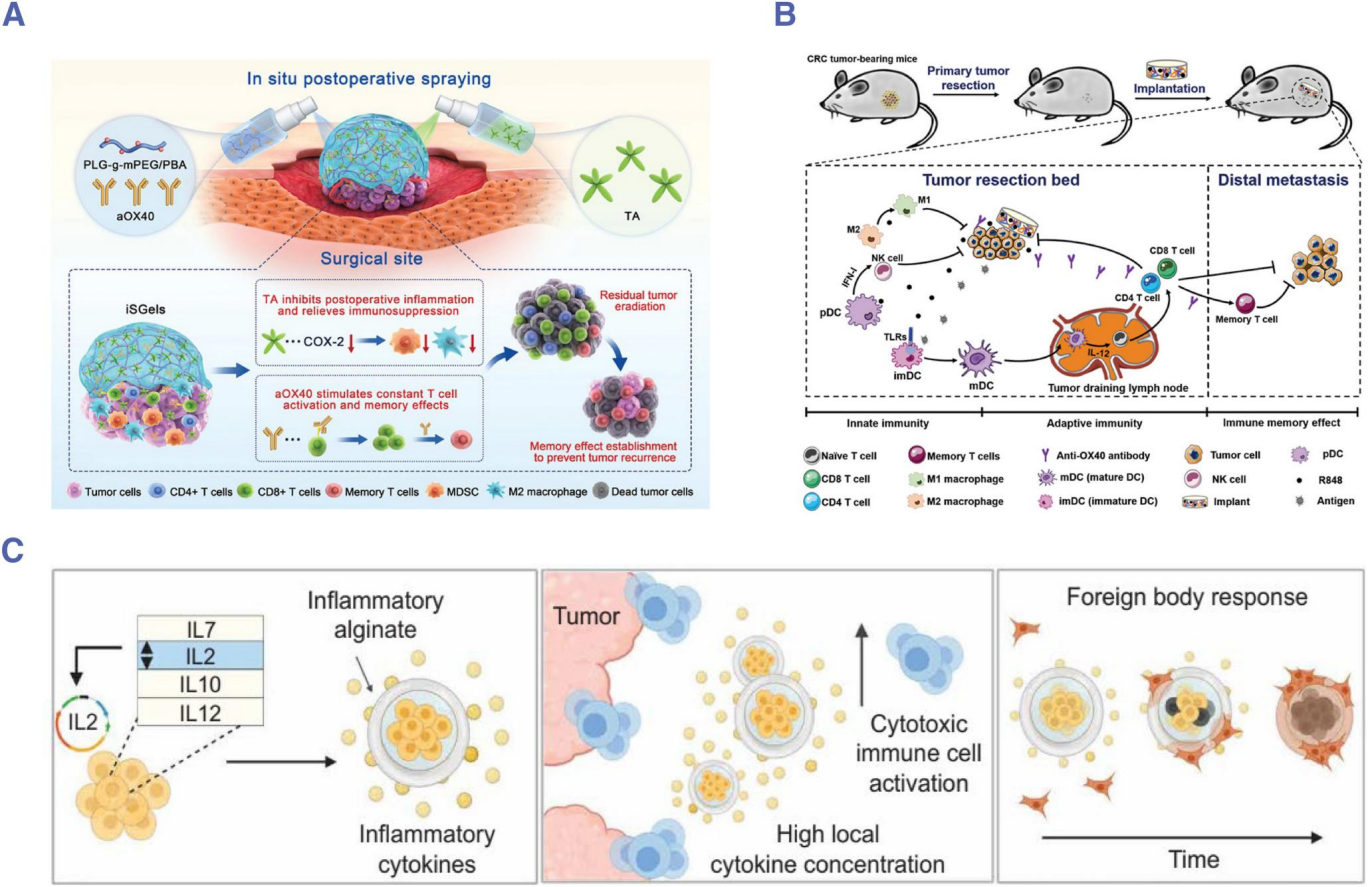
(A) Schematic illustration of the preparation and mechanism of the in situ‐sprayed gel for postoperative treatment of colorectal cancer.^22^ (Reprinted with permission. Copyright 2021, John Wiley and Sons.) (B) Schematic illustration of the biopolymer implant loaded with resiquimod and OX40 antibody for preventing postoperative recurrence and metastasis of colorectal cancer.^75^ (Reprinted with permission. Copyright 2021, John Wiley and Sons.) (C) Schematic illustration of the implantable cytokine factory.^24^ (Reprinted under the terms of the Creative Commons CC BY license. Copyright 2022, The Authors, published by American Association for the Advancement of Science).

As we all know, surgery is the leading choice for the treatment of PSM. However, recurrence and metastasis are usually common after surgery due to the local and systemic immune suppression caused by surgery.^27^ Hence, regulating the immune microenvironment of the peritoneal cavity and activating systemic antitumor immunity are essential for the postoperative treatment of abdominal tumors. Chen and colleagues reported a kind of in‐situ‐sprayed gel (iSGel) loaded with OX40 antibody (aOX40) for postoperative treatment of colorectal cancer.^22^ The iSGel is formed instantaneously after cross‐linking tannic acid with poly (L‐glutamic acid)‐G‐methoxy poly (ethylene glycol)/phenylboric acid, which has strong adhesion ability and can adhere to abdominal wall tissues (Figure 4A). Tannic acid can improve the immunosuppressive microenvironment after surgery. The aOX40 can activate T cells and enhance the effect of T cells. The antitumor effect of the immunotherapeutic gel was verified in a mouse peritoneal tumor model. Their results showed that the constructed system could inhibit the growth of peritoneal metastasis, produce an immune memory effect, and inhibit tumor recurrence. Furthermore, Chen and his team constructed a polymer implant by crosslinking 4‐arm poly (ethylene glycol) amine (4‐arm PEG‐NH_2_) and oxidation degree dextran (ODEX) by Schiff base reaction containing resiquimod and OX40 antibody for postoperative immunotherapy of colorectal cancer.^75^ Resiquimod is a small molecular Toll‐like receptor 7/8 agonist, which can stimulate the secretion of type I interferons (IFNs) of innate immune cells.^76^ This system can sequentially activate innate and adaptive immunity and produce an anti‐tumor effect with immune memory (Figure 4B). However, they only verified the antitumor effect of the implant in a mouse subcutaneous tumorigenesis model, and its effect on PSM remains unclear. Subsequently, Chen and his team optimized the implant with ODEX and 8‐arm polyethylene glycol amine. Oxaliplatin and resiquimod were loaded into the implant, which was used for the treatment of peritoneal metastasis. Their results suggested that the implant activated the antitumor immunity and cured 75% of mice with peritoneal metastasis.^77^ In summary, Chen et al. provided a new strategy for intraperitoneal immunotherapy.

Proinflammatory cytokines have been approved by the FDA to treat metastatic kidney cancer and melanoma. Infusions of high‐dose cytokines are necessary for effective cytokine therapy, but they can also lead to systemic side effects and resistance. To overcome these limitations, Omid Veiseh and his colleagues developed a cytokine delivery platform composed of genetically modified human retinal epithelial cells (ARPE‐19) encapsulated in alginate microparticles that produce the cytokine interleukin‐2 (IL‐2).^24^ Through the intraperitoneal administration, this platform enables spatial and time‐controlled dose adjustment for effective immunotherapy of peritoneal cancer without significant systemic toxicity (Figure 4C).

In general, intraperitoneal immunotherapy is promising for PSM treatment. However, current immunotherapy drugs have some problems, such as off‐target toxicity, short treatment time, and poor immunogenicity. Developing drug delivery systems for co‐delivery of immune drugs and synergistic drugs is a practical solution to overcome the defects of immunotherapy.

### 4.3 Gene therapy

Traditionally, gene therapy is a treatment that corrects or replaces a pathogenic gene with a normal or wild‐type gene. At present, the concept of gene therapy has been dramatically expanded. Any treatment that adopts the methods and principles of molecular biology at the nucleic acid level can be called gene therapy. Gene mutations are common in tumors; therefore, targeted gene therapy is expected to inhibit the progression of tumors or cure tumors.^23^, ^78^

**FIGURE 5:**
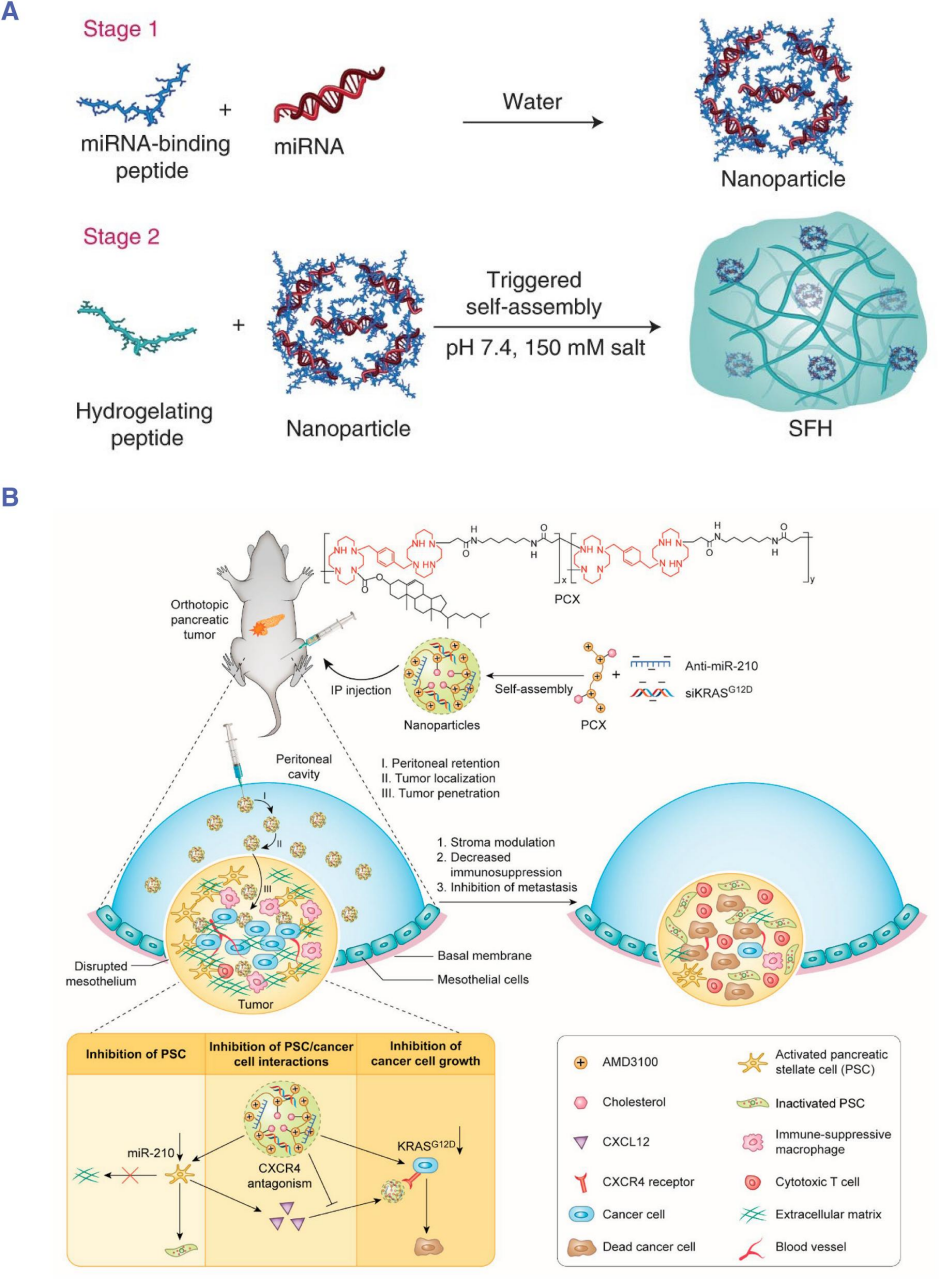
(A) Illustration of the surface‐fill hydrogel loaded with miRNA nanoparticles for the treatment of peritoneal and pleural mesothelioma.^23^ (Reproduced with permission. Copyright 2021, Springer Nature.) (B) Illustration of polymeric nanoparticles for the treatment of metastatic pancreatic cancer.^78^ (Reproduced with permission. Copyright, 2020, American Chemical Society).

Schneider and his colleagues constructed a surface‐fill hydrogel (SFH) loaded with miRNA nanoparticles for the adjuvant treatment of peritoneal and pleural mesothelioma, which is a less common type of PSM (Figure 5A).^23^ Specifically, the miRNA nanoparticles are prepared with negatively charged miRNA and positively charged miRNA‐binding peptides. Nanoparticles are encapsulated in the network of the hydrogel formed with the self‐assembled peptide. Then, miRNA‐215 and miRNA‐206 were encapsulated into SFH. miRNA‐215 can promote the proliferation of mesothelioma by activating the p53‐dependent apoptosis pathway, and miRNA‐206 can interfere with the cell cycle of mesothelioma. Local delivery of miRNA‐215 and miRNA‐206 can inhibit preclinical models of peritoneal and pleural mesothelioma, and a single dose can produce a sustained therapeutic effect. Similarly, Xie et al. described simple cholesterol‐modified polymeric CXCR4 antagonist nanoparticles for codelivery of negatively charged anti‐miR‐210 (to inactivate stroma‐producing pancreatic stellate cells) and positively charged siKRASG12D (to kill pancreatic cancer cells) (Figure 5B).^78^ These nanoparticles can effectively inhibit metastatic pancreatic cancer after intraperitoneal injection.

### 4.4 Combination therapy

#### 4.4.1 Chemotherapy combined with immunotherapy

Immunotherapy or chemotherapy alone has a limited effect on PSM. Recent studies have shown that drugs such as doxorubicin and oxaliplatin can induce immunogenic cell death in some tumors, resulting in the release of tumor antigens and the activation of the antitumor immune response.^79^, ^80^ Chemotherapy combined with immunotherapy has a synergistic effect in treating peritoneal metastasis.

**FIGURE 6:**
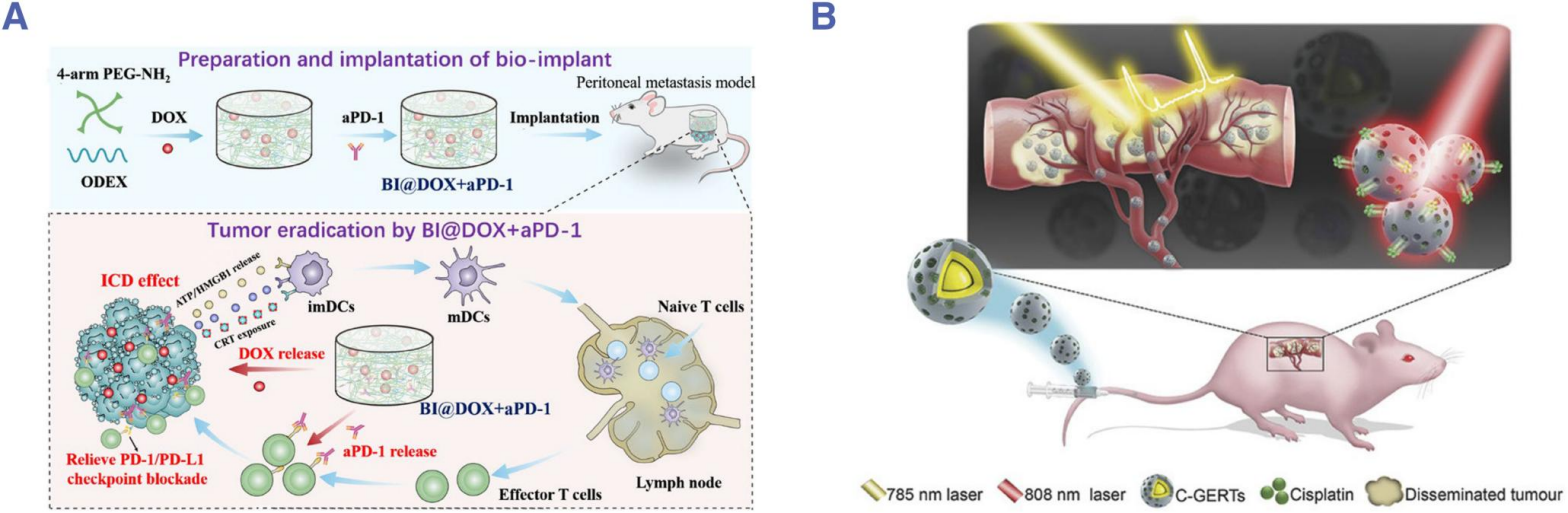
(A) Illustration of the biodegradable bio‐implant for synergistic immunotherapy and chemotherapy.^81^ (Reprinted with permission. Copyright 2020, American Chemical Society.) (B) Illustration of the system with functions of Raman‐guided tumor detection and chemo‐photothermal treatment for disseminated peritoneal microtumors.^82^ (Reprinted with permission. Copyright 2018, John Wiley and Sons).

Si et al. constructed a degradable bio‐implant loaded with doxorubicin and programmed death‐1 protein (PD‐1) monoclonal antibody based on the Schiff's base reaction between ODEX and 4‐arm PEG‐NH_2_ to treat peritoneal metastasis of CT26 tumor cells in mice (Figure 6A).^81^ Their results showed that the bio‐implant‐loaded drugs could degrade gradually in the abdominal cavity of mice and release drugs, resulting in a tumor suppression rate of 89.7%. Besides, the combination of the two drugs synergistically enhanced tumor suppression.

#### 4.4.2 Chemo‐photothermal therapy

Zhang et al. developed multifunctional surface‐enhanced Raman scattering nanoprobes composed of core–shell gold nanoparticles and a mesoporous silica layer.^82^ Raman reporters and cisplatin are loaded into the system. The cisplatin‐loaded gap‐enhanced Raman tags (C‐GERTs) can be used to detect occult peritoneal metastasis of ovarian cancer (Figure 6B). Raman signals help to find small nodules of peritoneal metastasis.

Gold nanoparticles have a photothermal effect. Then, chemo‐photothermal therapy is used to eliminate metastasis lesions. This strategy significantly prolonged the overall survival of mice with PSM.

### 4.5 Other therapies

#### 4.5.1 Photodynamics therapy

**FIGURE 7:**
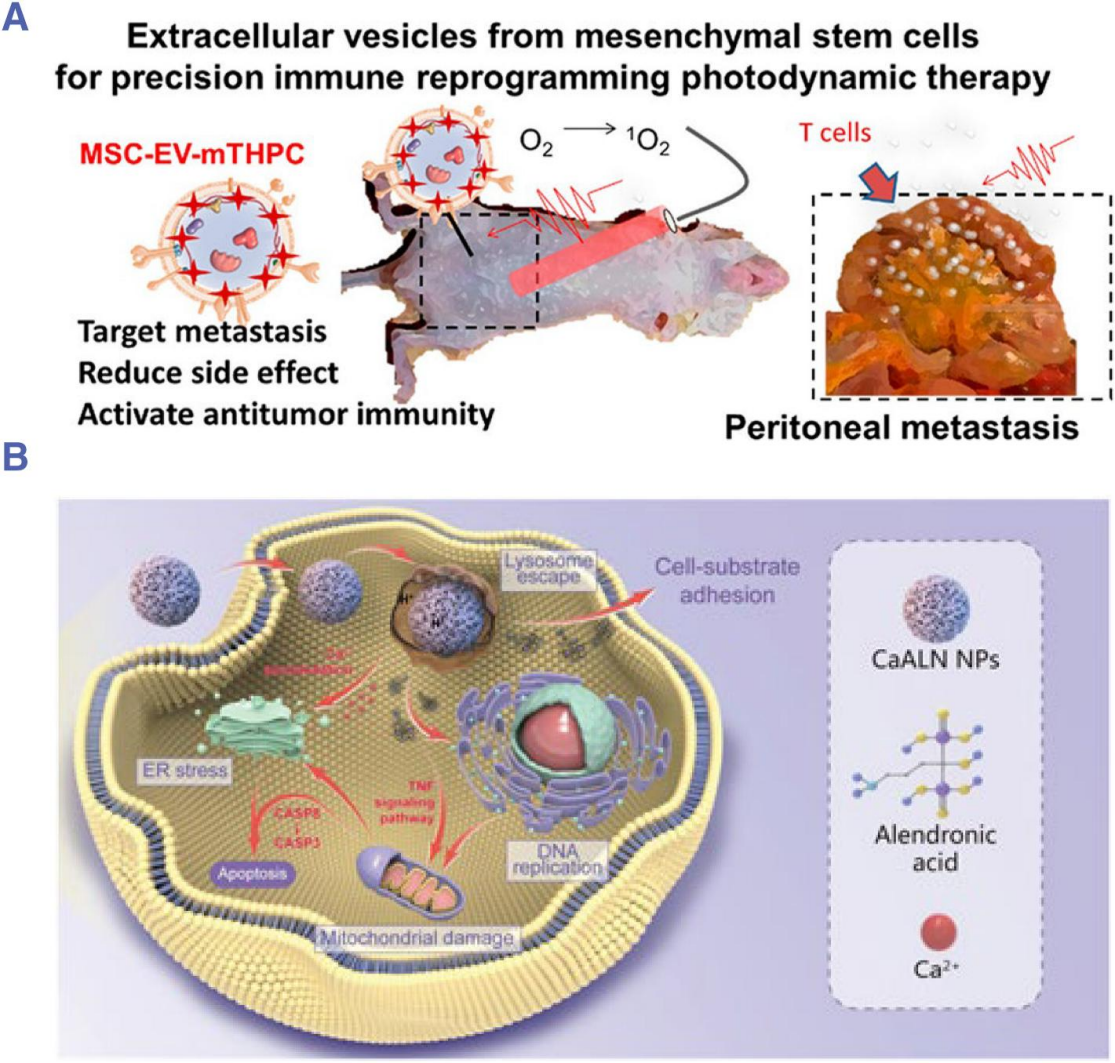
(A) Diagram showing the mechanism of MSC‐EV‐mTHPC against CT26 tumors.^85^ (Reprinted with permission. Copyright 2021, American Chemical Society). (B) Diagram showing the mechanism of action of CaALN against SKOV3 tumors.^86^ (Reprinted under the terms of the Creative Commons CC BY license. Copyright 2022, The Authors, published by John Wiley and Sons).

Photodynamic therapy (PDT) in treating peritoneal metastasis is limited clinically due to the low selectivity of photosensitizers (PS) for tumors and severe adverse reactions.^83^ Gazeau and colleagues used extracellular nanovesicles (EVs) derived from mesenchymal stem cells (MSCs) as carriers of the fourth‐generation immunoactive photosensitizer (PS) mTHPC. EVs can selectively target peritoneal metastasis for their inherent tropism to the tumor niche,^84^ which enhanced the killing effect of PDT at tumor sites (Figure 7A).^85^ Moreover, PDT‐induced tumor cell death further stimulated antitumor immune responses. However, the penetration of PDT is limited, and for the complex anatomy of the abdominal cavity, photodynamic‐based treatment may not be used for clinical translation.

#### 4.5.2 Targeting iron metabolism

Previous studies have shown that the demand of iron in tumor cells is much higher than that in normal cells, and iron plays an important role in supporting the proliferation and metastasis of tumor cells.^87^, ^88^ Hence, targeting tumor iron metabolism to inhibit tumor growth is a potential treatment strategy.^89^ Zhao et al. using a bisphosphonate with metal chelating function, alendronate (ALN), designed a CaALN iron nanochelator to kill cancer cells by the synergistic effect of reducing iron and accumulating calcium (inducing intracellular ROS elevation) in tumor cells (Figure 7B).^86^ Their results showed that CaALN nanoparticles could accumulate in tumor tissues and cause a significant inhibition of tumor growth and ascites formation in a mouse model of intraperitoneal dissemination with SKOV3 cells.

## 5 THERANOSTIC STRATEGIES IN PSM

**FIGURE 8:**
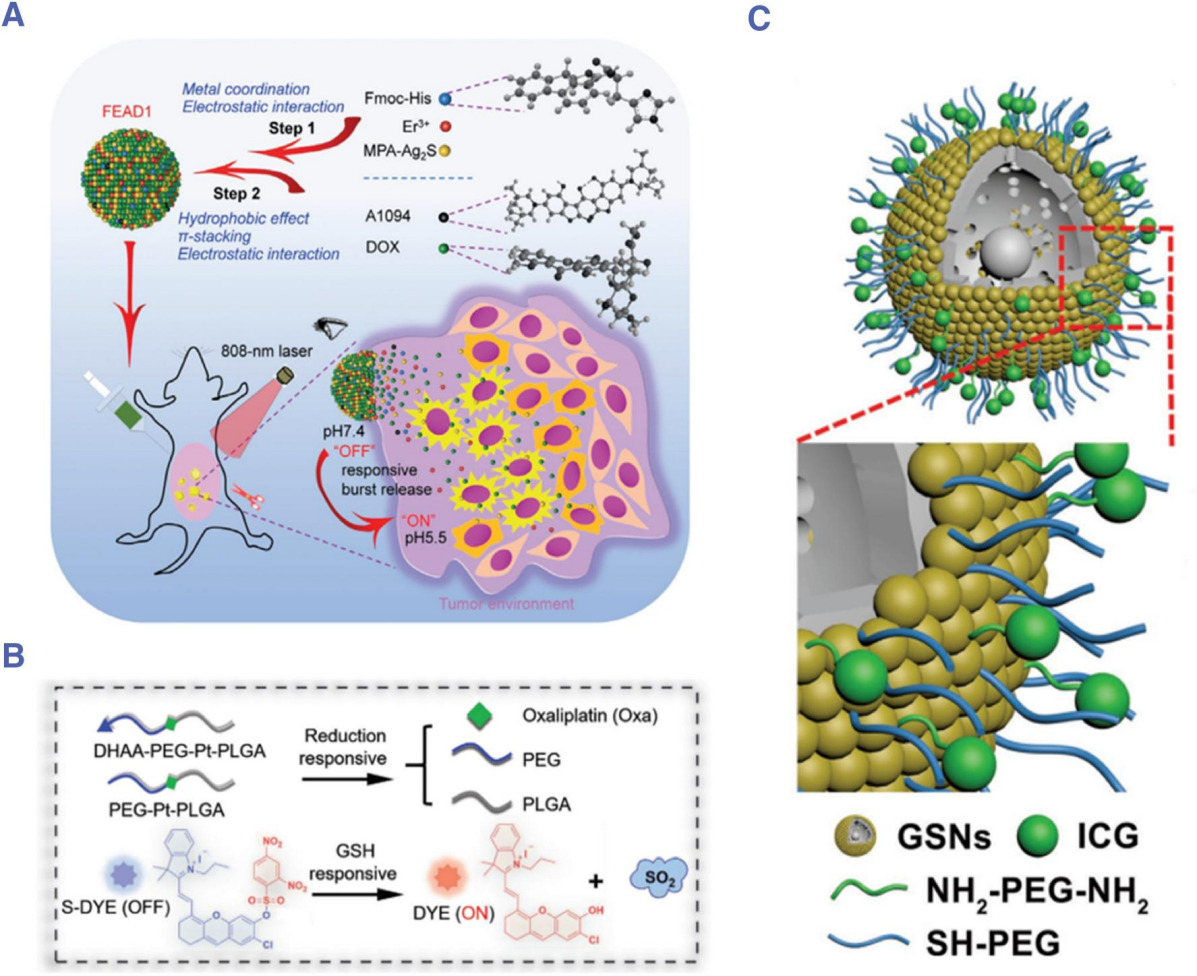
(A) Illustration of a nanotheranostic system and its capability to accurately detect and treat peritoneal metastasis.^90^ (Reprinted with permission. Copyright 2020, John Wiley and Sons.) (B) Illustration of a versatile theranostic platform for tumor tracking and photothermal‐enhanced chemotherapy for colorectal peritoneal metastasis.^91^ (Reprinted under the terms of the Creative Commons CC BY license. Copyright 2021, The Authors, published by John Wiley and Sons.) (C) Illustration of photothermal adjunctive cytoreduction to treat gastric peritoneal metastasis.^92^ (Reprinted with permission. Copyright 2018, John Wiley and Sons).

The theranostic system can make the dual functions of imaging diagnosis and therapy, which is a very promising direction for the management of PSM. Wang and colleagues developed a NIR‐II theranostic system for the management of peritoneal metastasis in a xenograft model with MDA‐MB‐231 cells (human breast cancer cells), which can be activated by the tumor microenvironment.^90^ More specifically, they prepared the nanotheranostics system (FEAD1) by self‐assembling nanoparticles with the peptide FMOC‐His, mercaptopropionic acid functionalized Ag_2_S (MPA‐Ag_2_S) quantum dots (QDs), doxorubicin, and near‐infrared absorber A1094 (Figure 8A). They found that FEAD1 was presented in the NAR‐II fluorescence off state in healthy tissues due to the interaction of Ag_2_S QDs‐A1094, while DOX remained in stealth mode. In delivering FEAD1 to tumors, the acidic tumor microenvironment triggers dissociation by disrupting the metal coordination bond and hydrophobic interaction of the system. The release of A1094 turns on Ag_2_S fluorescence to illuminate the tumor, accompanied by the release of DOX in the tumor tissue, thus achieving precise tumor treatment.

Sun et al. developed a theranostic system for colorectal peritoneal metastasis, which can be used for tumor tracking and photothermal‐enhanced chemotherapy (Figure 8B).^91^ Specifically, they linked PEG and PLGA with oxidized oxaliplatin (IV) to produce amphiphilic micelles. The micelles are then modified with targeted dehydroascorbic acid (DHAA), which allows the micelles to target the tumor cells. Then, they wrapped the quenched NIR photosensitizer (S‐DYE) in micelles. When the system reaches the reducing tumor microenvironment, the micelles disintegrate and release free oxaliplatin and S‐DYE. Under the action of GSH, S‐DYE changes to an active state to realize hyperthermia‐enhanced chemotherapy and real‐time imaging.

In addition, Wang et al. used gold nanoshells conjugated with indocyanine green for photothermal adjunctive cytoreductive surgery.^92^ Specifically, the nanoparticles (GSNs) are composed of 120 nm mesoporous silicon and 30 nm gold nanoshells (Figure 8C). GSN conjugated PEG was used to improve the stability of the nanoparticle, and conjugated ICG was used for NIR fluorescence imaging. This system can realize not only image‐guided CRS but also adjunctive photothermal therapy. The results of the study showed that this strategy could effectively eliminate peritoneal metastasis of gastric cancer in mice and prolong their overall survival.

## 6 CONCLUSION AND PROSPECTS

PSM has very poor prognoses, and there are many difficulties in the diagnosis and treatment. In recent years, studies have suggested that functionalized biomaterials play a vital role in the diagnosis and treatment of PSM. Through a literature review, we found that researchers focused on fluorescence‐guided surgical navigation materials and the construction of biomaterial‐based drug delivery systems.

Fluorescence‐guided surgical navigation helps remove peritoneal metastases indistinguishable from the naked eyes.^11^ Current studies mainly focus on the tracers of ovarian cancer peritoneal metastasis, which may be due to its relatively specific targeted receptors. Developing fluorescent probes that can target other pathologic types of PSM is an urgent issue. Besides, immuno‐PET can precisely detect tumors preoperatively,^93^, ^94^ which is a very promising research direction.

The applications of drug delivery systems have addressed some of the challenges of intraperitoneal chemotherapy; however, some problems remain challenging. Firstly, the inadequate drug penetration depth is a significant challenge due to high interstitial fluid pressure caused by imperfect lymphatic vessels and interstitial fibrosis.^95^ Secondly, although intraperitoneal chemotherapy should maintain a long local residence time, it should also improve the targeting ability of drugs, as drugs concentrated in the peritoneal cavity for a long period may cause peritoneal toxicity. Therefore, future research on the ideal drug delivery system for intraperitoneal therapy should take tumor characteristics and effective tissue penetration into account. Besides, current studies mainly focused on PSM that has developed into visible peritoneal tumors. Strategies to hinder intraperitoneal transport and mesothelial adhesion of tumor cells, and invasion of the submesothelial layer are vital to prevent the occurrence of PSM, which are also potential research directions.

Moreover, the safety of functional biomaterials for the diagnosis and treatment of PSM calls for our attention. The safety of biomaterials is dependent on several factors, including their biocompatibility, toxicity, and immunogenicity. Biocompatibility refers to the ability of the biomaterial to interact with human tissues without causing adverse effects. Toxicity refers to the potential of the biomaterial to cause harmful effects due to the release of toxic substances. Immunogenicity refers to the potential of the biomaterial to trigger an immune response in the body. To ensure the safety of biomaterials, rigorous testing is required before clinical use. This includes in vitro laboratory testing and in vivo animal testing. Before undergoing clinical trials in humans, biomaterials must be extensively characterized to ensure their safety and efficacy.

## AUTHOR CONTRIBUTIONS

Hui Wang, Xiusen Qin, and Junwen Ye conceived the idea and designed the review. Xiusen Qin drafted the manuscript. All the authors discussed and revised the review.

## CONFLICT OF INTEREST STATEMENT

All authors declare that there are no competing interests.
